# An evaluation of COVID-19 transmission control in Wenzhou using a modified SEIR model

**DOI:** 10.1017/S0950268820003064

**Published:** 2021-01-08

**Authors:** Wenning Li, Jianhua Gong, Jieping Zhou, Lihui Zhang, Dongchuan Wang, Jing Li, Chenhui Shi, Hongkui Fan

**Affiliations:** 1 National Engineering Research Center for Geoinformatics, Aerospace Information Research Institute (AIR), Chinese Academy of Sciences, Beijing 100094, China; 2 University of Chinese Academy of Sciences, Beijing 100049, China; 3 Zhejiang-CAS Application Center for Geoinformatics, Jiaxing 314199, China; 4 School of Geology and Geomatics, Tianjin Chengjian University, Tianjin 300384, China

**Keywords:** COVID-19, emerging infectious disease, SEIR modelling, virus transmission simulation

## Abstract

In December 2019, the first confirmed case of pneumonia caused by a novel coronavirus was reported. Coronavirus disease 2019 (COVID-19) is currently spreading around the world. The relationships among the pandemic and its associated travel restrictions, social distancing measures, contact tracing, mask-wearing habits and medical consultation efficiency have not yet been extensively assessed. Based on the epidemic data reported by the Health Commission of Wenzhou, we analysed the developmental characteristics of the epidemic and modified the Susceptible-Exposed-Infectious-Removed (SEIR) model in three discrete ways. (1) According to the implemented preventive measures, the epidemic was divided into three stages: initial, outbreak and controlled. (2) We added many factors, such as health protections, travel restrictions and social distancing, close-contact tracing and the time from symptom onset to hospitalisation (TSOH), to the model. (3) Exposed and infected people were subdivided into isolated and free-moving populations. For the parameter estimation of the model, the average TSOH and daily cured cases, deaths and imported cases can be obtained through individual data from epidemiological investigations. The changes in daily contacts are simulated using the intracity travel intensity (ICTI) from the Baidu Migration Big Data platform. The optimal values of the remaining parameters are calculated by the grid search method. With this model, we calculated the sensitivity of the control measures with regard to the prevention of the spread of the epidemic by simulating the number of infected people in various hypothetical situations. Simultaneously, through a simulation of a second epidemic, the challenges from the rebound of the epidemic were analysed, and prevention and control recommendations were made. The results show that the modified SEIR model can effectively simulate the spread of COVID-19 in Wenzhou. The policy of the lockdown of Wuhan, the launch of the first-level Public Health Emergency Preparedness measures on 23 January 2020 and the implementation of resident travel control measures on 31 January 2020 were crucial to COVID-19 control.

## Introduction

In December 2019, initially isolated cases of pneumonia due to infection with a novel coronavirus progressed to an outbreak in Wuhan, Hubei, China. On 20 January 2020, the National Health Commission of the People's Republic of China (NHC) confirmed that this novel coronavirus could spread from person to person [1]. On 24 January, during the Lunar New Year, the high travel volume and mass gatherings further increased the risk of virus transmission. To combat public health emergencies from local transmission, on 23 January 2020, all public transport and air travel access in Wuhan was suspended, 11 million residents were quarantined, and Lunar New Year celebrations were cancelled in many cities in China. To block the potential routes of virus transmission, residents were asked to minimise travel and wear masks in accordance with scientific evidence of efficacious virus control. Under a series of control measures, the spread of the virus was quickly managed. Since 16 February, the number of new cases in China has gradually decreased.

During the epidemic emergency, the Chinese government implemented some of its most stringent control measures. However, the epidemic in China has now entered a manageable stage. We need to review the outbreak process of this epidemic, further analyse the effectiveness and sensitivity of various control measures, and quantitatively evaluate the role of the implemented prevention and control policies. At present, many scholars are studying the effect of public health interventions on epidemic control from different perspectives [2–5]. The Susceptible-Exposed-Infectious-Removed (SEIR) model is one of the most popular mathematical models of infection that can be used to evaluate the effectiveness of various control measures [6], and it plays a key role in making many public health decisions. Godio *et al*. [7] applied an SEIR epidemiological model to the COVID-19 outbreak in Italy. Tian *et al*. [8] quantitatively evaluated COVID-19 transmission and the effects of control efforts on the epidemic in China.

The incubation period and the time from symptom onset to hospitalisation (TSOH) are important simulation parameters in the SEIR model. Some studies use a fixed value to replace these normally time-varying parameters. Some papers set the incubation period to approximately 5 days [7, 9–11]; clearly, the incubation period in a population is a range, and it is inaccurate to represent it with a fixed value. In our model, the randomness of the viral latency is taken into account, and the normal distribution function is used to determine the incubation period in the patients. In fact, the incubation period of COVID-19 is typically between 0 and 14 days. Similarly, the TSOH is a time-dependent parameter that is closely related to the given medical treatment and transportation methods. Detailed individual data have been published for Wenzhou, allowing us to simulate these parameters more accurately. In addition, many studies approach the region studied by the SEIR model as a closed system; they only consider the internal spread of the virus and ignore any cases imported from other regions. Therefore, we chose to improve the SEIR model by introducing parameters associated with imported cases [7, 12, 13].

To evaluate the effectiveness of epidemic prevention measures, many studies have focused only on the comprehensive evaluation of all prevention and control measures or on the role of only one measure. Lai *et al*. [9] proposed an SEIR model that incorporated human mobility to simulate the development of the epidemic in China and to evaluate the effect of non-pharmaceutical interventions, but they did not consider the impact of population segregation and the local implementation of unique measures on the model. Chinazzi *et al*. [14] simulated the spread of COVID-19 in China and abroad based on the global mobility model, and they verified the impact of the Wuhan travel ban. Maier and Brockmann [15] used the simpler Susceptible-Infected-Removed (SIR) model to study the comprehensive effect of China's prevention and control measures on curbing the spread of the novel coronavirus.

During the outbreak stage of the epidemic in China, the prevention and control methods implemented in Wenzhou were of great concern to people all around the country. Many migrant workers travel to Wenzhou, leading to many imported cases, and the number of people returning to work and school was estimated to reach 2.07 million. Additionally, many locals were infected during the early stage of the outbreak. In the face of this challenge, the Wenzhou health authorities instituted a series of positive and effective measures to control the epidemic. On 15 March 2020, the Health Commission of Wenzhou reported that the number of local cases had been reduced to zero, and the zero growth of local cases for 14 consecutive days indicated that the epidemic had been basically brought under control. The relevant departments have made great progress through epidemiological investigations, obtaining relatively complete individual survey data to provide a database that could be useful if the virus begins to spread again. Therefore, the aim of this paper is to model the transmission dynamics of COVID-19 based on a modified SEIR model and movement control measures in Wenzhou. By exploring the mechanism behind epidemic prevention and control in Wenzhou City, we quantitatively evaluated the control measures implemented during the epidemic, providing a reference for Chinese and global epidemic prevention and control.

## Study case and data

### Study area

**Fig. 1.**
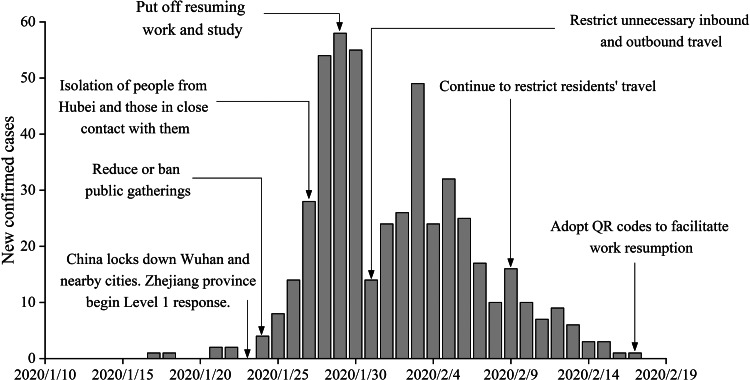
Number of cases showing the onset of illness among the 504 confirmed cases of COVID-19 and the control measures introduced in Wenzhou, China.

Wenzhou is located in Southwest China, with a total area of 11 612.94 km^2^ and a permanent population of 9.3 million. As of 18 March 2020, a total of 504 cases of infection have been reported, with approximately 0.054 infections per thousand people. The Health Commission of Wenzhou released individual data on 473 patients, of whom 196 had a history of travel before the onset of the disease, and 174 had a history of travel to Hubei, while imported cases accounted for approximately 36.8% of the total number of infections. With so much at stake, the government implemented a series of effective measures, achieving zero growth after 25 days (Fig. 1).

### Data sources

**Fig. 2.**
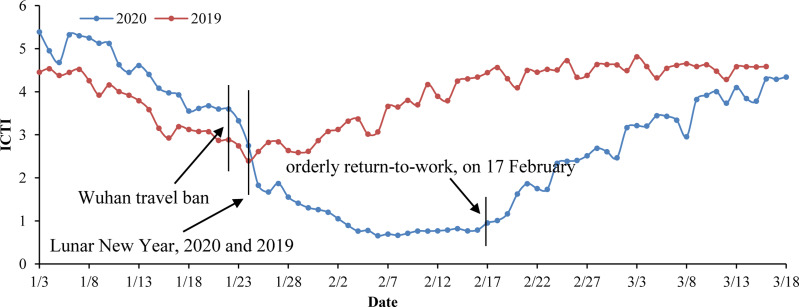
Intracity travel intensity in Wenzhou.*ICTI travel intensity refers to the ratio between the number of urban travellers and the resident population of the city (from the Baidu Migration Big Data Platform, https://qianxi.baidu.com).

1. Daily COVID-19 report data from Wenzhou. These data were obtained from the official website of the Health Commission of Wenzhou (http://wjw.wenzhou.gov.cn). The statistical characteristics primarily include daily new confirmed cases, cured cases and deaths. Individual data characteristics include the sex, age, address, history of case exposure, date of symptom onset, date of visitation and history of travel to Hubei. As of 13 March 2020, a total of 504 cases were confirmed, consisting of one death and 503 cured cases; of these, the individual data pertaining to 473 cases were published. In terms of sex, men were affected more often than women (Table 1). The numbers of men and women infected were 249 and 224, respectively, accounting for 52.6% and 47.4% of the total cases. The ages of the infected population ranged between 2 and 93 years. The median age of the patients in the outbreak was 48 years old. The number of people in the 40–60 age group was the largest. Patients over 40 years of age accounted for 71.9% of the total. The majority of infected patients were middle-aged and elderly. The male patients were primarily 40–60 years of age. The female patients were primarily 31–60 years of age. Compared to those of the male patients, the ages of the female patients were younger and more widely distributed.
2. Intracity travel intensity (ICTI) data. The Baidu Map Migration Big Data platform can be used to download the Wenzhou ICTI data obtained during the outbreak in 2020 and during the same period in 2019. These data were used to assess the number of daily contacts on different dates [5, 16]. On 23 January 2020, the ICTI began to decline rapidly due to the Wuhan travel ban and the first-level Public Health Emergency Preparedness event. The daily ICTI from 3 to 17 February was <1, which is 79.4% lower than that of the same period in 2019. After 17 February, under the strict implementation of epidemic prevention and control measures, companies were allowed to resume work and production in an orderly fashion, and crowd activities gradually returned to normal levels. The working day curve is stable, while small troughs appear at the weekends (Fig. 2).

**Table 1.** Statistics on confirmed cases of different ages and genders in Wenzhou

Age	Numbers of males	Numbers of females
1–10	1	2
11–20	6	2
21–30	30	20
31–40	35	50
41–50	77	49
51–60	63	53
61–70	22	32
71–80	13	13
81–90	1	3
91–100	1	0

## Methods

### Modified SEIR model

Transmission dynamics models play an important role in studying the transmission speed and routes of infectious diseases and the effects of control measures. Common infectious disease models include SI, SIR, SIRS and SEIR [17–19]. The SEIR model is an extension of the standard SIR model for infectious disease suitable for those with a certain period of latency. The standard SEIR epidemiological model has four components: susceptible, exposed, infected and removed (including recovery and death). ‘Susceptible’ refers to people who are not infected but can easily become infected after contact with an infected person. ‘Exposed’ refers to asymptomatic people carrying the virus. ‘Infected’ refers to obviously sick people. ‘Removed’ refers to people who have recovered or died and are thus no longer involved in the infection process. In this paper, the SEIR model is modified in light of the known propagation characteristics of COVID-19 and government interventions.

**Fig. 3.**
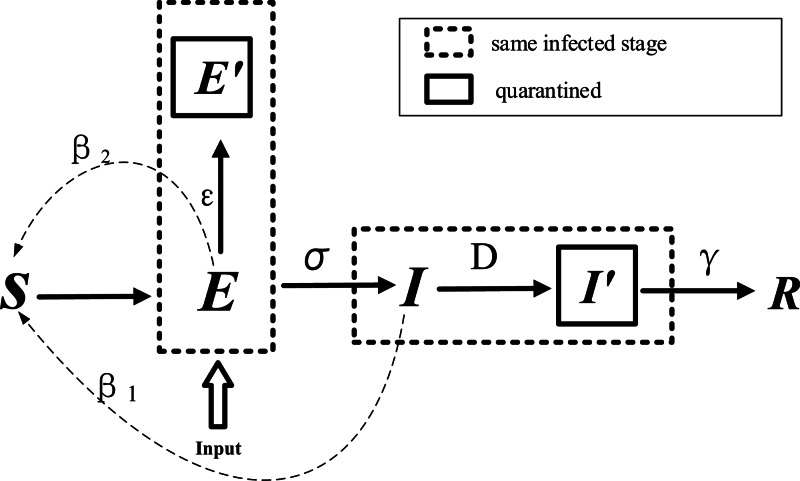
Modified SEIR model for COVID-19.

The modified model is shown in Figure 3 and Table 2. *E* refers to carriers of the virus. The model assumes that the input to the *E* block in urban areas is negligible compared to the total population; in other words, the total population remains unchanged. *ɛ* refers to the probability that a person with status *E* will be traced and isolated into *E*^′^ to limit their activities, while the remaining will perform their normal daily activities and contact 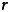 people. *β*_2_ is the probability that a susceptible person will become infected by an exposed person, and *D* refers to the number of days after onset that an individual is diagnosed and placed in isolation in the hospital for treatment. An infected person (*I*) has a probability of recovery and of producing antibodies to become rehabilitated or of treatment ineffectiveness and death (*R*). The modified SEIR propagation formula for COVID-19 virus is as follows:

1. An exposed person cannot transmit the virus to others in the standard SEIR model. However, COVID-19 shows infectivity during the incubation period [20]. Therefore, the infection probability *β*_2_ of the virus in the incubation period is introduced into the model.
2. We added the input parameter *E*_in_ for the exposed portion of the model during the early stage of the epidemic.
3. Contact tracing and isolation of confirmed patients is considered in the model. The probability of isolation is represented by *E*^′^ = ɛ × *E*. The value of *ɛ* is related to the intensity of the implementation of the tracing and isolation policy.
4. Because the infectivity and contact number of virus carriers are different during different periods, the parameters of infection probability and the daily contact number for exposed and infected persons are set separately [21]. *β*_1_ and *β*_2_ represent the infection probability from an infected person and an exposed person, respectively. *r* represents the average daily contacts made by free-moving persons. *E*^′^, *I*^′^ and *R* have been isolated. Assuming that the infection rate is set to 0, the number of contacts is no longer considered.
5. Because the symptoms of COVID-19 are similar to common influenza, and as a result of the lack of effective detection during the early stage of the outbreak, diagnosis is more difficult and extends the time of treatment, so the model increases the onset to hospital interval *D*, which is used to assess the efficiency of hospital testing and treatment; that is, hospitalised cases were admitted on *D* days after symptom onset.
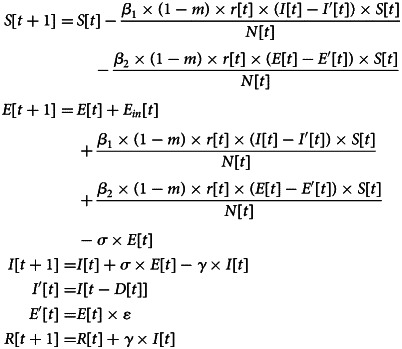

**Table 2.** Model parameters

Variable	Parameter
*β*_1_	Rate of transmission to susceptible people by infected people
*β*_2_	Rate of transmission to susceptible people by exposed people
*m*	Strength of protective measures
*r*(*t*)	Number of contacts per person per day, related to control policies
*D*[*t*]	Time from symptom onset to hospitalisation
*N*[*t*]	Total population in a region
*S*[*t*]	Number of susceptible people in a region
*E*[*t*]	Number of exposed people
*E*_in_[*t*]	Number of imported exposed people
*σ*	Incubation rate
ɛ	Quarantine rate
*I*[*t*]	Number of infected and unconfirmed people in a region
*I*^′^[*t*]	Number of infected and confirmed people in a region
*γ* = 1/*C*	Average rate of recovery or death, which depends on the average duration of infection
*R*[*t*]	Number of recovered or deceased patients
*A*(*t*)	Intracity travel intensity
*E*^′^[*t*]	Number of exposed and quarantined people

### Statistical analysis of epidemic data and evaluation of model parameters

According to the detailed individual data released by the Health Commission of Wenzhou, some parameters of the modified SEIR model can be obtained by statistical analysis. Parameters *β*_1_, *β*_2_, *m* and ɛ are obtained by fitting the model.

1. Analysis of epidemic prevention and control stage. According to the development of the epidemic and the implementation of intervention measures, virus transmission can generally be divided into three stages. During the first stage, the number of new infections will increase exponentially due to insufficient attention from the population. During the second stage, the number of people who travel will be reduced. During the third stage, the spread of the epidemic has been controlled, and the virus carriers are basically isolated in a restricted area. When all the exposed persons gradually develop symptoms and become infected persons, they are either ultimately cured or die.

Effective reproduction number (*R*_*t*_) changes caused by control measures. *R*_*t*_ is a variable related to infection interventions. It can reflect the effectiveness of external intervention measures and be used to judge trends in infectious diseases, and it can also be used as a reference for infectious disease risk management policies [22]. If *R*_*t*_ > 1, the number of cases will increase exponentially, suggesting that the prevention and control measures should be optimised and strengthened. When *R*_*t*_ < 1, the infectious disease will gradually disappear, and the current prevention and control measures will gradually manage the epidemic. *R*_*t*_ is commonly calculated based on a Bayesian probability estimation, which can be divided into two primary steps. The first step is to estimate the sequence interval distribution using known case data. For the second step, according to the input data and the posterior distribution of the sequence interval distribution obtained during the first step, the regeneration number can be estimated as a function of time [23, 24]. The probability of local cases is expressed as
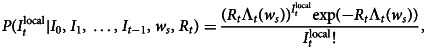
where 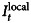 represents the number of new local cases on day *t*. *W*_*s*_ is the intergenerational time distribution. *R*_*t*_ is the reproduction number on day *t*. Λ_*t*_ is the total number of cases. Due to the direct calculation of *R*_*t*_ it is assumed that the regeneration number is stable over a period of time (*τ*). *R*_*t*_ can thus be computed as follows:
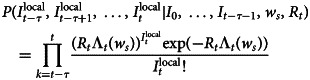
The R package EpiEstim is used to calculate the *R*_*t*_ value [23]. Before 23 January, *R*_*t*_ was >1, but a downward trend was evident, because it fluctuated between 2 and 4 before 17 January. From 17 to 22 January, the *R*_*t*_ fluctuated between 1 and 2. From 24 January to the peak of new daily cases (29 January 2020), the *R*_*t*_ continued to decrease. After falling below 1 on 30 January, it continued to show a downward trend. After 18 February, *R*_*t*_ stabilised below 0.1.

**Fig. 4.**
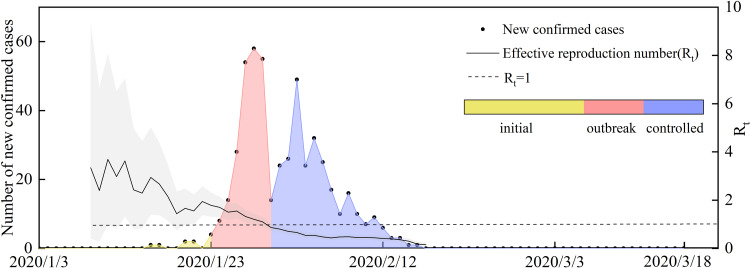
Number of new confirmed cases and *R*_*t*_ of local cases per day. 3 January, first confirmed patient enters Wenzhou; 23 January, Wuhan city travel ban; Zhejiang Province begin Level 1 response; 31 January, each family was limited to one person leaving the home every 2 days; and 18 March, no new local cases for 30 consecutive days.

We analysed the epidemic under the implementation of different prevention and control measures in Wenzhou (Fig. 4). The number of new confirmed cases was relatively flat before 23 January 2020. From 24 to 31 January 2020, the number of new cases showed exponential growth. After the implementation of a series of measures, such as travel restrictions, social distancing and resident travel control, the epidemic was controlled, and the *R*_*t*_ began decreasing rapidly. Therefore, the epidemic development in Wenzhou can be divided as follows. The first stage was from 3 to 23 January 2020, that is, the period from when the first patient entered Wenzhou to the first-level public health response in Zhejiang Province. During the second stage, from 24 to 31 January 2020, during the critical period of epidemic prevention and control, a large number of people in the exposed stage began showing symptoms, leading to a rapid increase in the number of newly diagnosed daily cases. The third stage was after 1 February 2020. The number of exposed patients no longer increased, and all the infected people were either cured or were deceased.

**Fig. 5.**
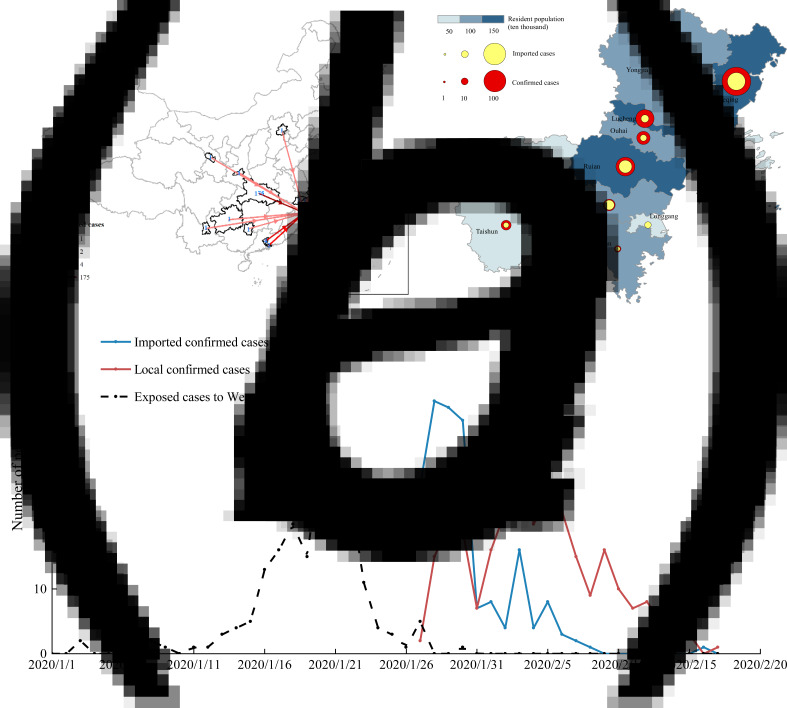
(a) Source distribution of imported patients in Wenzhou; (b) the background colour shows the population density, the size of the red circle indicates the total number of confirmed patients, and the size of the yellow circle indicates the number of imported confirmed patients; and (c) statistical plots of the daily number of patients and local patients and of the number of imported people.

**Fig. 6.**
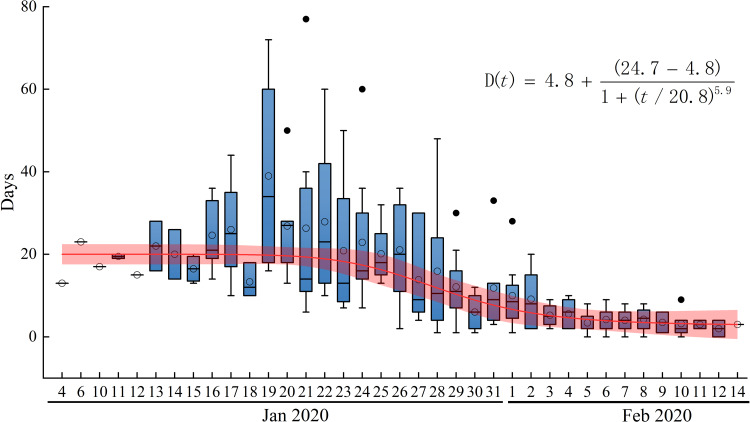
Time from symptom onset to hospitalisation and isolation in days. *D*(*t*) is the fitting function of the average.

**Fig. 7.**
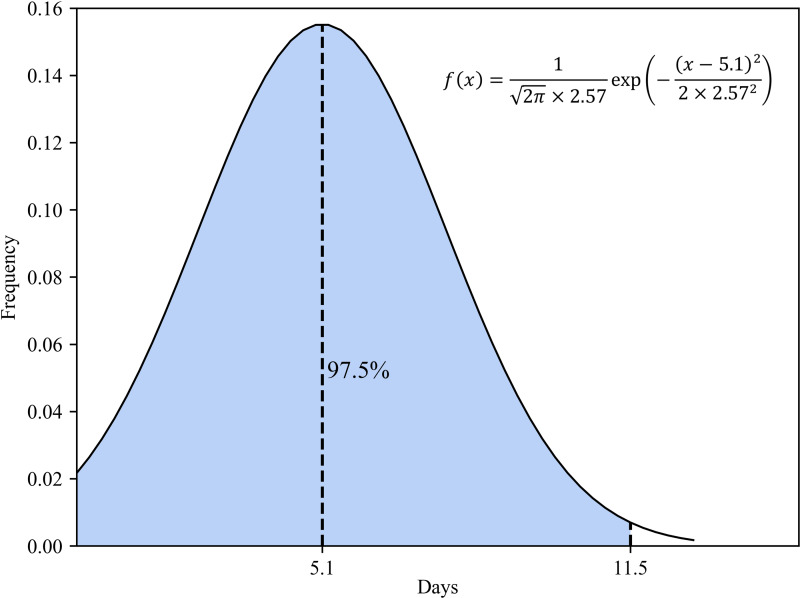
Normal distribution of the latency period.

1. We analysed the characteristics of the imported cases in Wenzhou. There were 504 patients in Wenzhou, including 473 patients with individual data, 196 imported from other regions and 176 with a travel history to Hubei Province. A map of imported cases from other regions is shown in Figure 5a. The distribution of imported cases within the counties and districts of Wenzhou is shown in Figure 5b. A plot of the daily incidence of imported and local cases and of the date that visitors entered Wenzhou is shown in Figure 5c. The peak number of individuals that visited in one day (the dotted line in Fig. 5c) was reached on 20 January. The peak dates of diagnosis for the imported cases were 28–30 January (blue line in Fig. 5c). At this time, the number of cases that entered Wenzhou was greater than the number of local cases. The virus spread primarily from 10 to 28 January, which was the best time to control the spread of the virus. Local infections (the red line in Fig. 5c) became the primary source of diagnosis after 1 February. After 3 February, the number of new local cases decreased rapidly.
2. Analysis of the TSOH. The TSOH ranged from 0 to 23 days; values of 2–10 days account for 76% of the data, and the average is 7.07 days. From the box diagram (Fig. 6), we see that before 22 January, the average TSOH was approximately 20 days. With investment in relevant testing equipment and improvements in detection [25–27], the TSOH began to noticeably decrease. The TSOH ultimately stabilised to between 3 and 5 days. A curve was fitted to the median of the TSOH (the red line in Fig. 6), which was used as the calculation of the daily time interval.
3. Acquisition of the daily contact number for exposed and infected persons. The number of daily contacts primarily depends on the population structure of the city, which in turn is related to its population density and economic development level. Therefore, in many studies, the number of daily contacts is directly proportional to the urban population density or the degree of travel. The number of contacts per person per day *r* = *a* × ICTI. Before prevention and control, the ICTI was between 4 and 5. The number of daily contacts per person (NDCP) performing normal activities is set as 12–15, and *a* can be calculated to be 3. The isolation personnel's personal activities are limited and protected, so the infection probability is set to be approximately 0. Therefore, the exposed (NDCP) can be ignored in Wenzhou.
4. The ratio of cured cases and deaths to the total number of confirmed cases per day is calculated according to the actual data, that is,
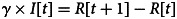
5. Methods of obtaining the transfer rate from exposed to infected patients. Many studies indicate that the incubation period of COVID-19 is 2–14 days, with an average of 5.1 days. In an experimental study by Stephen *et al*., the incubation period for each case was calculated based on a normal distribution with a mean value of 5.1 (95% CI 4.5–5.8 days), suggesting that 97.5% of latecomers would have symptoms within 11.5 days (Fig. 7). Considering the randomness of the incubation period, we used a normally distributed random number to represent the incubation period [11].
6. According to the population data of Wenzhou at the end of 2019 as released by the Zhejiang Provincial Bureau of Statistics, the permanent population of the city is 9.3 million. It is assumed that the initial susceptible population in Wenzhou is similar to that of its permanent residents. According to the epidemic data published by the Center of Disease Control (CDC) of Wenzhou on 23 January 2020, the initial infected person was someone who had lived in Wuhan for many years. He entered Wenzhou on 3 January and was diagnosed and hospitalised on 23 January. Therefore, 3 January is set as the starting date of the simulation, the initial value of the exposed person is 1, and the initial value of the infected person is 0.

## Results and discussion

### Reconstruction of COVID-19 spread based on the modified SEIR

**Fig. 8.**
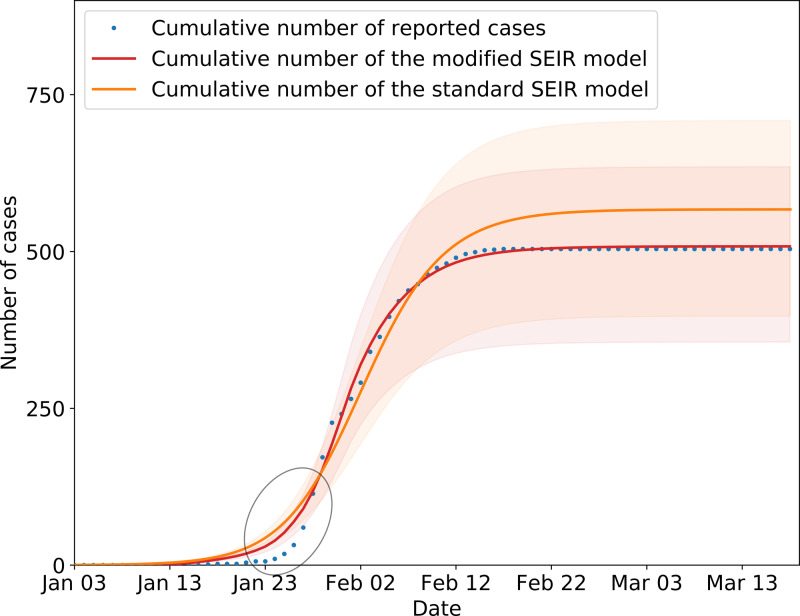
SEIR model simulation of epidemic control in Wenzhou. Shading indicates the inner 95% range of values.

In the SEIR model, changes in parameters *β*_1_ and *β*_2_ are related to climatic conditions and the virus itself. This paper assumes that the parameters remain unchanged in Wenzhou. Other parameters changed with changes in the control strategy, which includes public health prevention (*m*), the suspected case traces and isolation rate (ɛ), and the number of daily contacts (*r*) [28]. The number of daily contacts is obtained from the ICTI. Parameters *β*_1_, *β*_2_, *m* and ɛ must be obtained by solving a fitting model to obtain the optimal solutions, which are related to the research purpose and cannot be obtained from the known individual data. The performance of the model is tested by calculating the simulation results and the real value judgment coefficient *R*^2^. The closer the value of *R*^2^ is to 1, the higher fitting precision of the model. In this paper, the grid search method is used to solve the model parameters, and 100 solutions with the highest fitting parameter *R*^2^ are obtained, and then the average value of the parameters is calculated as the final value. The estimated parameters are shown in Table 3. Figure 8 shows the results of the model fitting and an estimation for the epidemic in Wenzhou from 3 January to 13 March 2020.
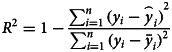
where *i* is the *i*th day, *y*_*i*_ is the real value of the cumulative cases, and 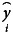 is the simulated value of the cumulative cases. 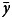 is the average value of the real value. The *R*^2^ of the modified model is calculated to be 0.997. In addition, we evaluated the fitting degree of the univariate linear regression between the predicted value and real value using the FTEST coefficient. The FTEST value of the predicted value and the true value in Microsoft Office Excel was 0.851, indicating that there was a significant linear correlation between the predicted value and the real value. The accuracy of the model is better, and it can be used for the subsequent evaluation of epidemic prevention and control measures. Additionally, we used the standard SEIR model to fit the epidemic data from Wenzhou, and the fitting results are shown in Figure 8. The *R*^2^ of the standard model-fitting results is 0.96 and the FTEST coefficient is 0.55. The modified SEIR was better than the standard SEIR.

**Table 3.** Parameters obtained from the fitting results

	*β*_1_ (0 − 0.1)	*β*_2_ (0–0.1)	*m* (0–1.0)	ɛ(0–1.0)
First stage, 1.3–1.23	0.027	0.02	0.1	0
Second stage, 1.23–1.31	0.027	0.02	0.5	0.65
Third stage, 1.31–3.18	0.027	0.02	0.5	0.85

The inflection point of the fit model was slightly different from the actual data, resulting in higher estimates than the reported number of cases. The primary reason for this difference was that first, it was difficult to detect and identify the virus during the pre-epidemic period; second, the population had a weak understanding of COVID-19, and they did not seek medical examination immediately even if they had symptoms; and third, the interval between patient diagnoses with symptoms during the early stage fluctuated greatly.

### Effects of different control measures

Four types of infection prevention and control measure were implemented in Wenzhou. Measure 1 – intercity travel restrictions and social distancing, which is represented by *r* in the model. Measure 2 – strengthen health protections, which is represented by *m* in the model. Measure 3 – contact tracing, which is represented by ɛ in the model. Measure 4 – early diagnosis and isolation, which is represented by *D* in the model. Based on the modified SEIR model, the sensitivity of the different measures in achieving epidemic prevention and control was assessed in two ways. First, the corresponding parameters of one measure were changed while the others were fixed; second, only one control measure was implemented at a time under certain conditions to verify the importance of the different prevention and control measures and their sensitivity in the model.

1. Six scenarios were created by controlling the corresponding parameters of one measure and keeping the other parameters unchanged to assess the sensitivity of each measure.

**Fig. 9.**
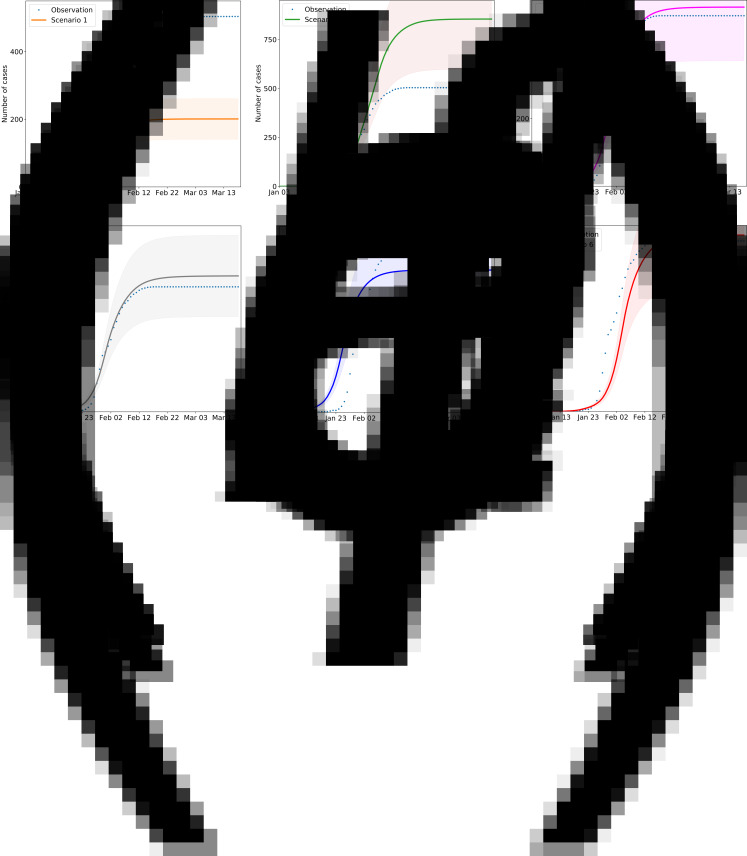
The impact of the timing and the absence of different measures on the number of infected people. The solid lines indicate the number of cases in the simulated scenario, the dotted lines indicate the officially confirmed number of cases; and shading indicates the inner 95% range. Scenario 1: Travel restrictions and social distancing measures were implemented 1 week in advance, namely, on 20 January 2020, after which the activity coefficient was simulated and the number of imported cases was set to 0. Scenario 2: Travel restrictions and social distancing measures are implemented 1 week late. Scenario 3: Susceptible groups have no protective measures; the efficiency of personal protective measures is set to 0. Scenario 4: Contacts of confirmed patients are not isolated. Scenario 5: The average time from onset to first medical visit and isolation is 3 days during the early stage of virus transmission. Scenario 6: The average time from onset to first medical visit and isolation is 8 days.

In the simulation analysis of the six scenarios (Fig. 9), 304 fewer persons were infected when measure 1 was instituted 1 week ahead of schedule, setting the value of *r* to decrease 1 week earlier in the model (Fig. 9a). Three hundred and fifty additional infections were predicted when a 1-week delay was implemented in the timing simulation of measure 1, setting the *R* value to delay for a week and start to decline in the model (Fig. 9b). With the hypothesis for scenario 3 setting the value of *m* to 0, we found that measure 2 has little impact on the model, which indicates that measure 2 can effectively slow virus transmission when implemented together with other measures (Fig. 9c), but the sensitivity is relatively lower than that of the other measures. In scenario 4, setting the value of ɛ to 0, 795 cases were expected without measure 3 (Fig. 9d). In the real environment, 6–7 days are required between symptom onset and hospitalisation. If the TSOH is reduced to 3 days, in other words, *D*[*t*] is set to 3 (Fig. 9e), the number of infected patients will be reduced by 16.7%, or approximately 84 cases according to the epidemic curve simulation. By contrast, if the TSOH is set to 8 days, *D*[*t*] is set to 8, and the number of infected patients will be increased by 10.2% (Fig. 9f). Therefore, we should increase our investments in medical equipment and improve the detection efficiency for the virus. We can conclude that the effects of these measures vary with one another, but all of them play a large role in epidemic prevention and control.

1. Only one measure is implemented to verify the need for certain prevention and control measures to manage epidemics.

**Fig. 10.**
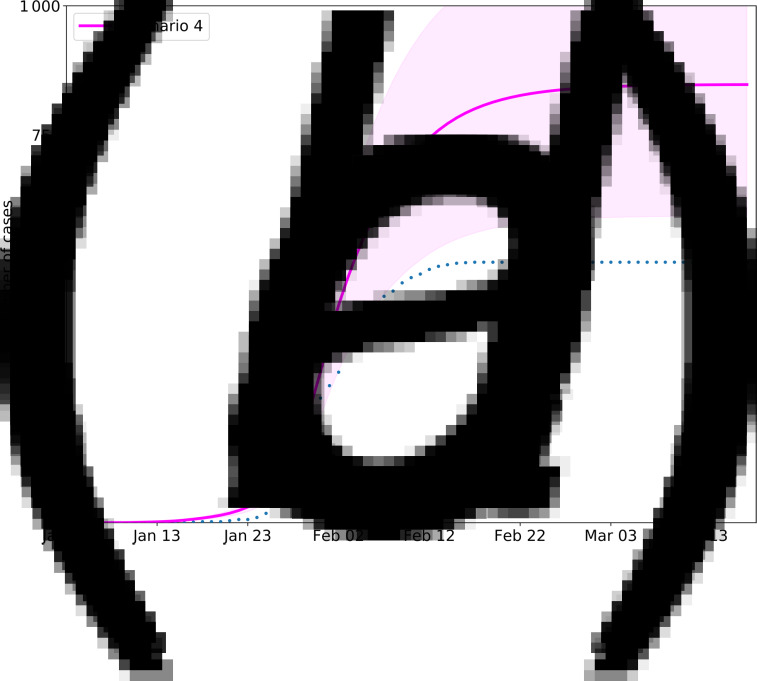
Scenario simulation results of travel restriction and social distancing. The solid lines indicate the number of cases in the simulated scenario, the dotted lines indicate the officially confirmed number of cases accordingly, and the shading areas indicate the inner 95% range. Scenario 1: No measures are taken. Scenario 2: Only travel bans and social distancing measures are implemented, with no follow-up measures. Scenario 3: Only mandatory masking and other personal protective measures can be taken. Scenario 4: Only close contacts are tracked, and the isolation density reaches 80%.

Figure 10a shows the predicted curve without any intervention. A comparison of the single-measure scenarios in Figure 10 shows that measure 1 (scenario 2) and measure 3 (scenario 4) can control the epidemic and cause the curve to flatten. However, the manpower and material resources required to perform the extensive contact tracing and monitoring during the 14 days of isolation needed to achieve these scenarios would be considerable. According to the official data on 8 March 2020, 14628 contacts were tracked. The travel ban and social distancing, including the most extreme phases of the shutdown, have caused at least a trillion CNY economic losses to society [29]. Measure 2 or 4 alone cannot curb the virus but can effectively slow its spread. Therefore, depending on the stage of the epidemic, this combination may be the most effective and feasible method to implement the necessary control measures.

1. Predicting the scale of an outbreak rebound and evaluating the control measures

**Fig. 11.**
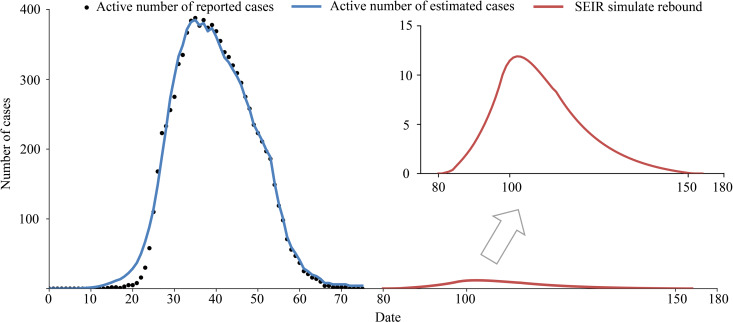
Simulation of an outbreak rebound, assuming that seven virus carriers were found on the first day of investigation.

At present, the COVID-19 situation is basically controlled, but the risk of another outbreak is large. The epidemic rebounded on 16 June 2020, due to a gathering at Xinfadi (Beijing Fengtai wholesale market). The emergency response level of the Emergent Events of Public Health in Beijing was adjusted from level 3 to level 2. Therefore, we should be aware that there are many challenges in preventing the epidemic from rebounding. For example, we can assume that there is a clustering epidemic in Wenzhou, and the initial number of people causing the epidemic is set to 1. When the epidemic is detected, the number of confirmed cases is 7. According to the model, 22 virus carriers are still free to move. If strict screening, isolation, home prevention and control are started immediately, the model predicts that the final number of infected people will be controlled to within 55. From the simulation results (Fig. 11), the possibility of large-scale outbreaks is small, but it is estimated that it will take 2 months to clear the infected persons, which would still cause great economic losses. Therefore, at present, the general population should still engage in active protection in the hopes of either avoiding an epidemic rebound or detecting and controlling it early.

## Conclusion

This study primarily analysed COVID-19 transmission dynamics and the effects of initial measures by governments. The epidemic was divided into three stages in the modified SEIR model. In comparison with the previous system dynamics studies, our research took the uncertainty of the incubation period and TSOH into account. Through a detailed analysis of individual data, some parameters of the model can be obtained accurately. We used the modified SEIR model to analyse and compare the cumulative number of cases quantitatively after the implementation of control measures for COVID-19 in Wenzhou.

The Wuhan lockdown cut-off the major transmission path of imported cases, and hence the number of imported cases dropped rapidly to less than 10 per day. However, before that, 175 cases had entered Wenzhou and caused a large number of transmissions, the mean value of *R*_*t*_ was at a high level between 2 and 4 and many local residents were infected. Furthermore, to reduce the transmission in an effort to delay and lower the epidemic peak, the Wenzhou government imposed restrictions on movement in local communities. On 23 January 2020, Wenzhou city launched the level 1 response, which banned public gatherings. Within 2 days, the ICTI dropped rapidly from 3.32 to 1.82. On 27 January, interprovincial and intercity passenger transport were suspended, the people coming from Hubei province were isolated for 14 days, and close contacts were tracked and isolated. On 29 January, the expressway into Wenzhou was closed, and the enterprises delayed the resumption of work and completely cut-off the case input. On 1 February, restriction on unnecessary inbound and outbound travel was imposed, and only one person from each family was allowed to go out for shopping every 2 days. These measures are aimed at reducing the spread of the disease in the population by preventing human-to-human contact, and it played a great role in curbing the spread of the virus. The real-time transmission coefficient of the virus dropped below 1 on 27 January, and the new cases reached their peak on 26 January. There were no new cases in the population from 15 to 29 February. All the above findings supported the effectiveness of the measures.

We tested the sensitivity of the model to the different control measures and evaluated the effectiveness and timing of four different non-drug interventions. If lockdown and social distancing are delayed by one week, we expect an increase of 69%. It was found that the duration from illness onset to hospitalisation was long, and it took at least 5 days for 52% of the patients to be hospitalised (Fig. 6). If this time was reduced to 3 days, the number of inpatients might be greatly reduced. Health-protective behaviours and close contact tracing have contributed to disease control. However, these approaches must be combined with other measures to contain the epidemic. We found that social distancing alone, as implemented in Wenzhou during the outbreak, is sufficient to control COVID-19, while health-protective behaviours cannot interrupt transmission on their own, although they can reduce the peak incidence and delay the epidemic.

It is evident from this study that the intensity and start time of intervention measures have a significant impact on the spread of COVID-19. The travel restrictions during the Wuhan City lockdown, social distancing and the level 1 response played a key role in curbing the epidemic in Wenzhou. Since identifying and isolating early cases of the disease is difficult, investing vast resources in improving the Covid-19 testing capacity is part of a containment strategy. Additionally, improving the isolation rate of asymptomatic close contacts and increasing the treatment rate of symptomatic infected persons are essentially as equally effective at controlling epidemic transmission. In addition, we suggest that people continue wearing masks and maintain social distancing in effect, to reduce the spread of COVID-19.

Before specific drugs and vaccines against COVID-19 are developed, blocking transmission and isolation have continued to be effective ways to prevent and control COVID-19. In preventing continuous and rapid increases in the number of infected people, more aggressive contact tracing, quarantining and isolation can effectively reduce or even block further virus transmission. Therefore, comprehensive non-drug intervention measures resulted in the strongest and fastest containment of the COVID-19 epidemic in Wenzhou, and the peak of the epidemic appeared approximately 1 week after their comprehensive implementation. In terms of future work, more variables could be taken into account, such as seasonal effects. This is because seasonal temperature changes could have a long-term influence on the spread of COVID-19 as referenced in [30]. Therefore, COVID-19 infection may be affected by the season and latitude in an unpredictable way. In addition, if we want to study transmission in a very large area, we need to consider the differences in social mixing patterns between asymptomatic infected people in urban and rural areas, which may lead to different transmission patterns. These research results are also important for comprehensively understanding the spread of COVID-19.

## Data Availability

The data used to support the findings of this study are available from the authors upon request.

## Supplementary material

For supplementary material accompanying this paper visit https://doi.org/10.1017/S0950268820003064. click here to view supplementary material
